# Improving quality of life in cancer patients with an app-based intervention (Mika-App): secondary results from a randomized controlled waitlist-trial in Germany

**DOI:** 10.1055/a-2826-5988

**Published:** 2026-04-08

**Authors:** Franziska Springer, Hannah Brock, David Kreilinger, Gandolf Finke, Jan Simon Raue, Anja Mehnert-Theuerkauf

**Affiliations:** 1 Department of Medical Psychology and Medical Sociology, Leipzig University, Leipzig, Germany; 2 Fosanis GmbH, Berlin, Germany

**Keywords:** cancer, quality of life, app-based intervention, randomized-controlled trial, Krebs, Lebensqualität, Digitale Unterstützungsangebote, Randomisiertekontrollierte Studie

## Abstract

**Objective:**

Psychological distress in cancer patients can be reduced through digital support tools, such as the Mika-App. Yet, the effect on various dimensions of quality of life (QoL) remains unclear. In a secondary analysis of a randomized controlled trial (RCT), we aimed to investigate the effect of the Mika-App on physical and mental QoL, and to explore potential moderators such as age, sex, and digital health literacy (DHL).

**Methods:**

Patients diagnosed with cancer within the past five years were recruited for this nationwide RCT in Germany. Participants were randomly assigned to either the intervention group (IG, access to the Mika-App), or the wait-list control group (CG, usual care). Online questionnaires were administered at baseline, 2, 6 and 12 weeks. Physical and mental QoL was assessed with the Short-Form Health Survey (SF-8). Linear mixed-effects models were used to test for changes in QoL over time between groups and to examine moderating effects. Analyses were conducted intention-to-treat (ITT) and per-protocol (PP).

**Results:**

A total of 218 patients (IG=99, CG=119) were included, with a mean age of 56 years, 61% being female. Patients in the IG demonstrated significant improvements in mental QoL over time compared to the CG (ITT: F(3, 623.46)=3.58, p=0.014; PP: F(3, 360.38)=5.54, p=0.001), with small-to-medium effect sizes between groups at the 6- and 12-week follow-ups (Cohens’ d=0.26–0.69). There was no change in physical QoL in both groups (ITT: p=0.90; PP: p=0.88). Age, sex, and DHL did not moderate the interventions’ effect on QoL (all p>0.05).

**Discussion:**

While physical QoL remained unchanged, mental QoL significantly improved. This reflects the app’s content features, mainly targeting self-management skills and psychological distress. The app appears to be equally beneficial across all age groups, for men and women. Since DHL was particularly high in our sample, possibly due to the predominantly online recruitment strategy, results on DHL need to be interpreted with caution. Further research is needed on the role of DHL in the efficacy of digital support tools in the context of routine clinical care.

**Conclusion:**

Digital support tools have the potential to improve QoL in patients with cancer. Integrating these tools into clinical care practice might provide valuable support for patients in need by addressing their specific symptoms.

## Zusammenfassung

**Ziel der Studie:**

Die psychische Belastung von Krebspatient*innen kann durch digitale Unterstützungsangebote wie die Mika-App verbessert werden. Unklar bleibt der Effekt auf verschiedenen Dimensionen der Lebensqualität. Ziel dieser Sekundäranalyse einer randomisierten kontrollierten Studie (RCT) war es, den Effekt der Mika-App auf körperliche und psychische Lebensqualität zu untersuchen und mögliche Moderatoren wie Alter, Geschlecht und digitale Gesundheitskompetenz (DHL) zu identifizieren.

**Methodik:**

Für diese deutschlandweite RCT wurden Patient*innen rekrutiert, bei denen innerhalb der letzten fünf Jahre Krebs diagnostiziert wurde. Teilnehmende wurden randomisiert der Interventionsgruppe (IG, Zugang zur Mika-App) oder der Wartelisten-Kontrollgruppe (CG, Standardversorgung) zugewiesen. Online-Fragebögen wurden zur Baseline sowie nach 2, 6 und 12 Wochen ausgefüllt. Die Lebensqualität wurde mit dem Short-Form Health Survey (SF-8) erfasst. Lineare gemischte Modelle prüften Gruppenunterschiede in der Veränderungen der Lebensqualität über die Zeit, als auch mögliche Moderationseffekte. Die Analysen erfolgten Intention-to-Treat (ITT) und Per-Protocol (PP).

**Ergebnisse:**

Insgesamt wurden 218 Patient*innen (IG=99, CG=119) eingeschlossen, mit einem Durchschnittsalter von 56 Jahren, 61% waren weiblich. Die IG zeigte im Vergleich zur CG eine signifikante Verbesserung der psychischen Lebensqualität über die Zeit (ITT: F(3, 623,46)=3.58, p=0,014; PP: F(3, 360,38)=5,54, p=0,001), mit kleinen bis mittleren Effektgrößen zwischen den Gruppen zum 6- und 12-wöchigen Follow-Up (Cohens‘ d=0,26–0,69). Die körperliche Lebensqualität unterschied sich nicht (ITT: p=0,90; PP: p=0,88). Es zeigten sich keine Moderationseffekte von Alter, Geschlecht und DHL (p>0,05).

**Diskussion:**

Während die körperliche Lebensqualität unverändert blieb, verbesserte sich die psychische Lebensqualität signifikant. Dies spiegelt die App-Inhalte wieder, die v. a. auf Selbstmanagement und psychischen Belastungssymptome abzielen. Die Wirksamkeit zeigt sich in allen Altersgruppen, bei Männern und Frauen. Die hohe DHL in unserer Stichprobe, möglicherweise durch die vorwiegend onlinebasierten Rekrutierung, erfordert eine Interpretation mit Vorsicht. Der Einfluss der DHL auf die Wirksamkeit digitaler Angebote muss im Rahmen klinischer Routineversorgung weiter untersucht werden.

**Schlussfolgerung:**

Digitale Unterstützungsangebote haben das Potential die Lebensqualität der Betroffenen zu verbessern. Die Integration in die klinische Versorgung bietet eine wertvolle Möglichkeit, Betroffene mit spezifischen Symptomen gezielt zu unterstützen.

## Introduction

Beyond the physical burden of a cancer disease, psychological distress, symptoms of anxiety or depression, and reduced quality of life (QoL) are common side effects among cancer patients 1 2 3 . The overarching goal of comprehensive cancer care is to support patients holistically with their individual symptoms and needs 4 . As demonstrated by large systematic reviews and meta-analyses 5 6 7 of psycho-oncological interventions, such as psychotherapy, psychoeducation, or relaxation, are effective in reducing distress and QoL in patients with cancer. The number of individuals living with cancer, however, increases rapidly 8 , which is largely attributable to advances in diagnostics and medical treatments, as well as an aging population. Access to supportive services is limited by organizational, and patient-related barriers 9 . As a result, there is a widening supportive care gap in the provision of adequate and long-term care for patients with cancer. Digital tools, which are highly flexible and scalable, are therefore increasingly becoming an integral part of oncological care. Systematic reviews and meta analyses have proven the beneficial effect of digital tools on patient-reported outcomes 10 11 12 .

We have recently investigated the efficacy of the digital therapeutic “Mika-App” on psychological distress in a nationwide randomized controlled trial (RCT) with a waitlist control group in Germany 13 . Mika is an app-based intervention that provides a broad range of psychoeducational, symptom monitoring, and self-management support. All content is tailored to the patients’ individual symptoms and needs. In our primary analyses we have demonstrated the efficacy of the app in reducing symptoms of distress, anxiety, depression, and fatigue 13 . However, we did not observe a change in global QoL, which may in part be explained by the single-item QoL measurement tool used in our primary analysis 14 . This may have been insufficient to capture nuanced changes of QoL. Previous research has shown that while global QoL often remains stable in cancer patients, specific domains, such as social functioning or symptom-related burden, tend to be more sensitive to change 15 . This observation may be explained by the “disability paradox” 16 , whereby individuals facing a distressing disease still report good QoL due to psychological adaptation over time and a response shift. In contrast, specific physical and psychological symptoms may not be subject to the same level of adaptation.

When examining health outcomes in patients with cancer, existing socioeconomic health inequities 17 and the digital divide 18 raise important questions about the efficacy of digital support tools across diverse patient groups. Age and digital health literacy (DHL), i. e. the ability to find, understand, and apply health-related information from electronic sources 19 , have been emphasized as potential moderating factors for the efficacy of digital support tools 20 21 . The results of an interview-based study of people with low-literacy compared to population surveys demonstrated that patients with lower income, lower levels of education, or older age face greater barriers to using and benefiting from digital support tools 20 .

In summary, it remains unclear how the Mika-App affects specific dimensions of QoL and whether patient-related factors or DHL moderate these effects. This secondary analysis of a nationwide RCT in Germany therefore aimed to (1) evaluate the efficacy of the Mika-App in improving QoL dimensions, assessed with a well-validated tool that measures physical and mental QoL, and (2) identify patient-related factors that might moderate the effect of the app on QoL.

## Methods

### Study Design and Participants

In a nationwide unblinded waitlist RCT, we evaluated the efficacy of an app-based intervention (Mika-App) on patient-reported outcomes. Patients were randomized to either the intervention group (IG) using the Mika-App during the study period, or the wait-list control group (CG) receiving usual care. Usual care comprises all healthcare options cancer patients usually receive in Germany (i. e. oncologist visits, social or psycho-oncological counseling). Assessments were completed at baseline (t0), 2 weeks (t1), 6 weeks (t2), and 12 weeks (t3) using self-report questionnaires. After completing the 12-week follow-up, the CG also received access to the Mika app. The study was approved by the Ethics Committee of Leipzig University (404/21-ek) and was registered at the German Clinical Trials Register (DRKS00026038).

### Recruitment and Procedure

Between September and November 2021, patients were recruited via social media (Facebook and Instagram), emails to cancer support groups throughout Germany, and direct outreach from a participant pool at the University Medical Center Leipzig. Eligibility was assessed via phone, during which the app’s name was withheld to prevent contamination of the CG, as the app is freely available. Eligible individuals received study information via email and were required to upload a physician’s letter confirming their cancer diagnosis. Following informed online consent, participants were randomized and completed online baseline questionnaires before being informed of their group assignment. IG participants received access to the app after completion of the baseline assessment with a brief standardized phone introduction. Questionnaires were administered online using LimeSurvey, with automated email reminders. Data collection was completed in March 2022.

### Randomization and Data Monitoring

Participants were randomized 1:1 using permuted block randomization (block size=4). The allocation list was concealed until assignment. Due to the nature of the intervention, blinding was not feasible. To maintain data quality, study staff contacted participants via phone after each assessment point to collect missing data, clarify questionnaire items, and offer technical assistance. Missing responses were documented during these calls reading missing questions verbatim and documenting the participants’ responses.

### Intervention

The Mika-App is a digital therapeutic providing tailored support to cancer patients of all tumor types. The app was developed by Fosanis GmbH in collaboration with researchers at Charité Berlin, University Medical Center Leipzig, and the National Center for Tumor Diseases Heidelberg. It includes three modules ( Fig. 1 ): Check-Up (distress and symptom tracking), Discover (educational content on cancer-related topics), and Journeys (interactive resource-activating training). The content is based on evidence-based interventions including mindfulness-based stress reduction (MBSR), acceptance-commitment therapy (ACT), cognitive-behavioral therapy (CBT), and physical activity guidance. All content in the app is expert-reviewed (e. g. oncologists, psychologists, nutritionists) and tailored through an artificial intelligence (AI) algorithm based on cancer diagnosis, treatment phase, and app usage behavior. For a more detailed description of the apps’ content, see previous publications 13 22 .

**Fig. 1:**
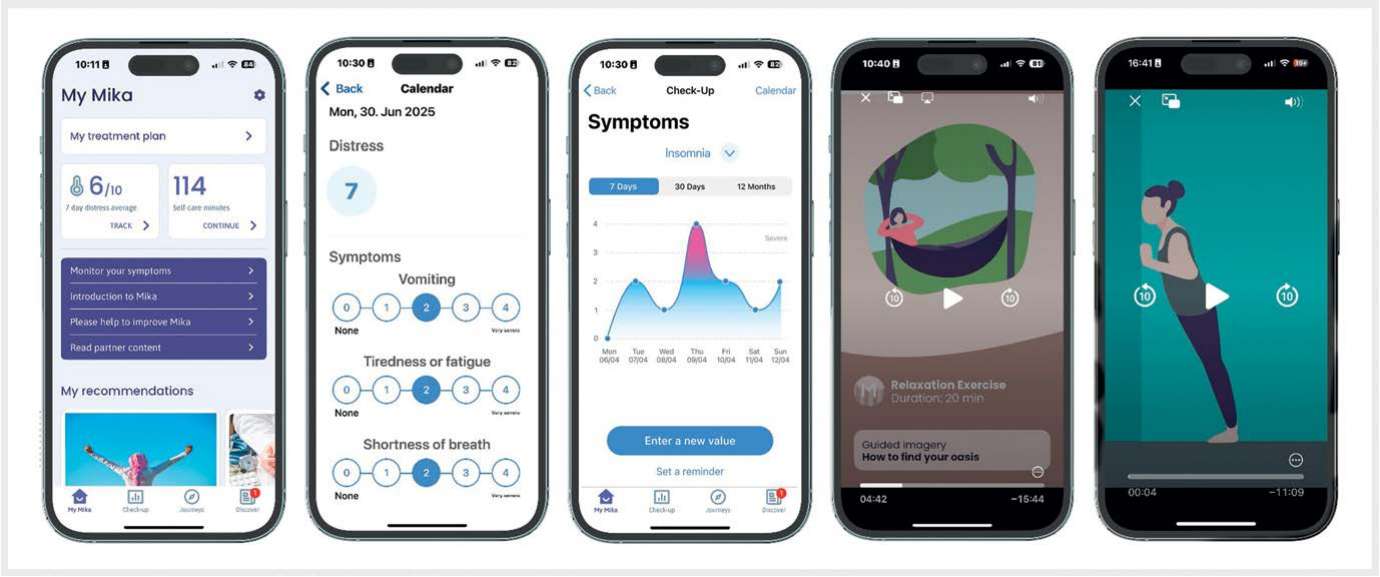
Screenshots of the Mika-App.

Participants in the IG were encouraged to use the app at least three times a week but could freely choose the frequency of use and its content.

### Outcome Assessment

QoL was measured using the 8-item Short-Form Health Survey (SF-8) 23 . It provides two summary scores, one for mental QoL (mental component score, MCS) and one for physical QoL (physical component score, PCS). Both scores are standardized ranging from 0 to 100, with higher values indicating better perceived mental or physical QoL.

The eHealth Literacy Scale (eHEALS) 24 was used to assess DHL. The 8-item self-report measure assesses perceived skills in finding, evaluating, and applying electronic health information to health problems. All items are rated on a 5-point Likert scale ranging from 1 (strongly disagree) to 5 (strongly agree), with total scores ranging from 8 to 40. Higher scores indicate better DHL. The eHEALS is widely used and demonstrates good reliability and validity across diverse populations 25 .

Sociodemographic and medical information (age, sex, cancer type, date of diagnosis) were assessed through submission of a physician’s letter. We further assessed if patients received additional psychotherapeutic support during the study via a telephone interview.

### Statistical Analysis

All analysis was conducted in an intention-to-treat (ITT) population, including all randomized participants, as well as per-protocol (PP), including participants with complete questionnaire data, with no psychotherapeutic support during the study, no app usage before the study, and for the IG minimum app usage behavior during the study (at least once every four weeks during the first eight weeks). Baseline differences between the IG and CG as well as study completers and dropouts were tested using independent samples t-test and Chi-square test.

To examine QoL changes over time between the IG and CG, separate linear mixed-effects models were computed to model trajectories across four time points (t0-t3) for QoL subscales MCS and PCS. Linear mixed-effects models were used as they appropriately handle missing outcome data due to dropout under the Missing at Random (MAR) assumption. All models were specified with random intercept, fixed effects for group (IG vs. CG), time (t0-t3), as well as their interaction (group×time). All models were adjusted for age and sex. Assumptions of normality of residuals, homoscedasticity, and linearity were assessed through visual inspection of residual plots. Follow-up post hoc comparisons of the estimated group means at each measurement point were conducted with pairwise contrasts and effect sizes Cohen’s d (small=0.2, medium=0.5, large=0.8). To explore the influence of potential moderators on the effect of the intervention on QoL, three-way interaction terms (group×time×moderator) were added to the linear mixed models for sex (men vs. women), age (<65 vs.≥65 years) and DHL (low vs. moderate/high).

Analyses were conducted in R statistics software (version 4.3.1). Linear mixed-effects models were conducted using the *lmer* function from the lme4 package. All statistical tests were two-tailed, with a significance level of 5%.

## Results

### Sample Characteristics

During the three-month recruitment period, 517 individuals were screened, with 321 meeting eligibility criteria. Of these, 248 provided informed consent (response-rate 77%) and were randomized to either the IG or CG. Participants without verified cancer diagnosis (n=30) were excluded, resulting in an ITT sample of 218 (IG=99, CG=119). The PP analysis included 124 participants (IG=50, CG=74) after excluding 94 participants mainly for reasons such as concurrent psychotherapeutic support, or prior use of the Mika-App. For a detailed study flow-chart, see previous publication 13 . Baseline sociodemographic and medical characteristics of the ITT population are displayed in Table 1 . Participants were on average 56 years old, 61% were female. The mean time since diagnosis was 25 months. The most common cancer types were breast (34%) and hematologic cancers (28%), with 9% being diagnosed with a relapse. DHL was moderate to high in most of the patients, with 10% in the IG and 21% in the CG having low DHL.

**Table:** **Table 1** Baseline sociodemographic and medical sample characteristics of the intention-to-treat (ITT) sample ( *N* =218).

	Intervention ( *n* =99)			Control ( *n* =119)	
Variable	*n*		%	*n*	%
Mean age, years (SD, range)	55 (11, 20–77)			58 (10, 35–76)	
Gender					
Women	65		65.7	67	56.3
Men	34		34.3	52	43.7
Cancer type					
Breast	38		38.4	36	30.3
Hematological	27		27.3	34	28.6
Lung	9		9.1	12	10.1
Urogenital	6		6.1	14	11.8
Gastrointestinal	7		7.1	11	9.2
Head-neck	3		3.0	4	3.4
Gynecological	3		3.0	3	2.5
Other	6		6.1	5	4.2
Relapse	7		7.1	12	10.1
Time in months since cancer diagnosis, mean (SD)	25 (18)			24 (16)	
Digital health literacy (DHL)					
Low	10	10.1		25	21.0
Moderate to high	89	89.9		94	79.0
Psychotherapeutic support during the study	15	15.2		17	14.3

Abbreviations: intention-to-treat (ITT), standard deviation (SD), digital health literacy (DHL)

Baseline characteristics were largely balanced between the IG and CG, i. e. for sex ( *p* =0.21), PCS ( *p* =0.94), and MCS ( *p* =0.33). However, IG participants were slightly younger than those in the CG ( *p* =0.02). Patients who dropped out of the study (n=37) did not differ from study completers (n=211) in age ( *p* =0.89) or sex ( *p* =0.23) but reported significantly worse MCS ( *p* =0.003) and PCS ( *p* =0.03) at baseline.

A descriptively analysis of app usage behavior was provided in our previous publication 13 .

### Quality of Life Over Time

Trajectories of mental QoL (MCS) and physical QoL (PCS), stratified by group, are displayed in Fig. 2 . For the MCS, the linear mixed model revealed a significant group×time interaction in the ITT population (F(3, 623.46)=3.58, *p* =0.014, Table 2 ), indicating that the trajectory of MCS scores differed significantly between the IG and CG. Post-hoc comparisons of MCS group means at each measurement point did not reach statistical significance ( Table 3 ). The PP analysis also showed a group×time interaction (F(3, 360.38)=5.54, *p* =0.001), with post-hoc comparisons showing better mental QoL in the IG compared to the CG at t2 (d=0.69, *p* =0.045) and a non-significant trend toward improvement at t3 (d=0.67, *p* =0.051) with medium effect sizes ( Table 3 ). There were no main effects of group and time in the ITT and PP analyses.

**Fig. 2:**
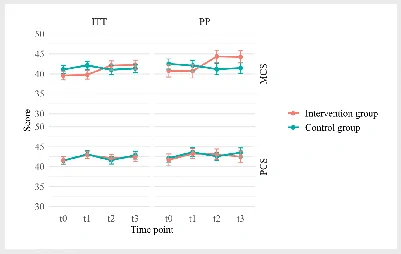
Mental and physical quality of life (QoL) over time, assessed using the SF-8, with a mental component score (MCS) and physical component score (PCS), in the intention-to-treat (ITT) and per-protocol (PP) populations.

**Table:** **Table 2** Changes in mental and physical quality of life (QoL) over time – Results from linear mixed models.

	Intention-to-treat		Per-protocol	
	F (df)	*p*	F (df)	*p*
**Mental Component Score (MCS)**				
Group	0.04 (1, 212.89)	0.836	1.02 (1, 120.00)	0.314
Time	0.65 (3, 623.60)	0.582	0.61 (3, 360.58)	0.608
Group×Time	3.58 (3, 623.46)	0.014*	5.54 (3, 360.38)	0.001**
**Moderation effects** ^**a**^				
Age	0.15 (3, 617.64)	0.930	0.51 (3, 354.13)	0.672
Sex	0.16 (3, 617.14)	0.923	0.38 (3, 354.29)	0.769
Digital health literacy (DHL)	0.35 (6, 605.11)	0.909	0.37 (6, 345.28)	0.895
**Physical Component Score (PCS)**				
Group	0.00 (1, 212.62)	0.965	0.28 (1, 119.68)	0.597
Time	2.34 (3, 622.55)	0.072	1.73 (3, 360.12)	0.158
Group×Time	0.19 (3, 622.44)	0.899	0.22 (3, 359.97)	0.880
**Moderation effects** ^**a**^				
Age	1.30 (3, 616.72)	0.272	3.24 (3, 353.77)	0.022*
Sex	0.15 (3, 616.29)	0.928	0.36 (3, 353.91)	0.779
Digital health literacy (DHL)	1.16 (6, 604.09)	0.326	1.31 (6, 344.85)	0.250

**Note** : Quality of life (QoL) was assessed using the SF-8, with a mental component score (MCS) and physical component score (PCS). Linear mixed models were specified with random intercept and fixed effects for group (IG vs. CG), time (t0–t3), and the interaction (group×time). Significant values are marked in bold (* *p<* 0.05, ** *p<* 0.005) ^a^ Age, sex, and digital health literacy (DHL) were included as moderators in the main linear mixed model analysis by incorporating three-way interaction terms (group×time×moderator)

**Table:** **Table 3** Post-hoc comparison of group means at each measurement point.

	Intention-to-Treat				Per-Protocol			
	t0	t1	t2	t3	t0	t1	t2	t3
**Mental Component Score (MCS)**								
Intervention, M (SD)	39.6 (11.0)	39.8 (10.8)	42.1 (11.0)	42.3 (11.4)	40.8 (10.9)	40.7 (12.2)	44.4 (10.9)	44.3 (10.7)
Control, M (SD)	41.1 (11.1)	42.1 (11.0)	41.0 (12.0)	41.4 (11.5)	42.5 (10.9)	42.1 (11.5)	41.2 (11.9)	41.5 (11.5)
Cohen’s d	–0.12	–0.24	0.26	0.28	–0.09	–0.05	0.69	0.67
*p*	0.615	0.308	0.283	0.250	0.784	0.881	0.045*	0.051 ^x^
**Physical Component Score (PCS)**								
Intervention, M (SD)	41.6 (9.4)	42.9 (8.5)	42.1 (9.4)	42.4 (10.1)	41.7 (10.4)	43.3 (9.0)	43.1 (9.4)	42.5 (10.0)
Control, M (SD)	41.5 (10.3)	43.1 (10.7)	41.6 (10.1)	42.9 (10.4)	42.2 (8.8)	43.6 (10.2)	42.6 (9.2)	43.6 (9.6)
Cohen’s d	0.00	–0.06	0.07	–0.06	–0.17	–0.17	–0.09	–0.30
*p*	0.999	0.841	0.794	0.826	0.657	0.648	0.815	0.431

**Note** : Quality of life (QoL) was assessed using the SF-8, with a mental component score (MCS) and physical component score (PCS). Estimated group means at each measurement point (t0 - t3) were compared using pairwise contrasts, and effect sizes were reported as Cohen’s d. Significant values are marked in bold ( ^x^ *p* <0.10, * *p* <0.05, ** *p* <0.005) Abbreviations: M=mean; SD=standard deviation

For the PCS, there were no significant group x time interactions in both the ITT (F(3, 622.44)=0.19, *p* =0.90) and PP analysis (F(3, 359.97)=0.22, *p* =0.88). Furthermore, no significant main effects were observed for group and time ( Table 2 ).

### Moderation Analysis

To examine whether participant characteristics moderated the intervention effect, three-way interaction terms (group×time×moderator) for age, sex, and DHL were added to the main models. Overall, no robust moderation effects were identified, neither for MCS nor PCS ( Table 2 ). Although the PP analysis showed a significant three-way interaction involving age (F(3, 353.77)=3.24, *p* =0.022), this effect was limited to t2 (results not shown).

## Discussion

This secondary analysis of a nationwide RCT provides further evidence of the efficacy of the Mika-App in improving mental QoL among patients with cancer. In contrast, physical QoL remained unchanged over time, with no differences between patients who used the app and the control group. The app’s effect on QoL outcomes was not influenced by age, sex, or DHL, suggesting that its benefits on mental QoL were consistent across these subgroups.

The observed improvements in mental QoL align well with the app’s focus on addressing psychosocial symptoms. The app offers psychoeducational information on side effects, monitoring of distress and symptoms to enhance a sense of control, as well as CBT-based modules on topics such as stress relief, acceptance, or sources of strength. The consistency of effects across both the ITT and PP analyses underscores the robustness of our findings. The small to medium effect sizes are comparable to those reported in other psycho-oncological interventions, including both offline formats 5 , as well as digital psycho-oncological interventions 10 11 26 27 28 .

In contrast, physical QoL remained largely unchanged. Although the app includes information on physical side effects and treatment guidelines, e. g. related to pain, nausea or cognitive impairments, these topics are not its primary focus. Patients particularly interested in these areas may have accessed relevant information, but this approach alone may be insufficient to lead to measurable improvements in physical QoL. It is important to note that this was not the primary goal of the Mika-App and more targeted interventions may be required. Existing app-based interventions for cancer patients vary widely in their content, e. g. physical exercises, mindfulness and relaxation, or symptom tracking, depending on their primary aims 29 . Digital interventions that provide tailored support for physical symptoms, by incorporating physical exercises and psychotherapeutic strategies to manage these symptoms, have demonstrated efficacy in improving outcomes such as pain 27 , fatigue 26 or cognitive impairments 28 .

Interestingly, we did not observe a moderating effect of age. A recent scoping review reports comparable or even higher mental QoL in older adults with cancer compared to younger cancer patients, whereas physical QoL tends to decline with advancing age 30 . Existing research on the role of age in the efficacy of digital interventions remains limited and yields mixed results. Some studies suggest that older patients may show even higher adherence to digital interventions compared to younger patients 31 , with patients agreeing that all age groups may benefit from digital support tools 32 . In contrast, other studies highlight greater barriers for older adults in accessing and benefiting from digital support tools 20 . There also remains a notable lack of digital interventions specifically tailored to the needs of older adults with cancer 33 . Ageist assumptions, such as lower digital literacy or less engagement among older adults, may further contribute to the underutilization of such tools 34 .

The absence of a moderating effect of DHL contrasts with previous findings suggesting that limited DHL can act as a barrier to the effective use of digital support tools 20 32 35 . One possible explanation lies in the characteristics of our sample: recruitment was conducted mainly online, which may have led to a relatively homogeneous study sample with sufficiently high DHL. Alternatively, the user-friendly design of the app may have mitigated potential barriers, allowing even those with basic DHL to navigate and benefit from the intervention. Further research is needed to explore these findings in more diverse, real-world clinical settings, where patients with a broader range of DHL levels are represented.

### Strengths and Limitations

The Mika-App offers comprehensive support that combines distress and symptom monitoring, psychoeducation, and a structured coping-skills program. This multifaceted approach addresses key components of psychological distress and QoL among cancer patients, aligning with evidence-based principles. The analysis by both ITT and PP principles strengthens the validity and robustness of our findings. The consistency of effects across these analytic approaches underscores the efficacy relating to mental QoL.

Some limitations are to be mentioned. First, the online recruitment strategy likely resulted in a sample that was particularly motivated, digitally literate, and proactive in seeking support. This introduces a selection bias and limits the generalizability of the findings to broader, more diverse patient populations, especially those with lower digital access or engagement. Future studies are needed in a more real-world clinical setting to validate our findings. Second, there is a potential bias due to dropouts. Since individuals experiencing higher distress may be more likely to discontinue participation, the true effect of the intervention might be underestimated. Third, the study did not collect data on participants’ socioeconomic status, education, or income. These factors are known to influence access to and benefits from digital health interventions, and the lack of data limits our ability to analyze potential disparities in the app’s efficacy across different socioeconomic groups. This, however, would be highly valuable to estimate the generalizability of our findings in diverse patient populations and to identify vulnerable subgroups. Fourth, app usage was assessed at the level of overall app use, and although adherence was rated as good in our primary analyses 13 , a more fine-grained evaluation of usage patterns, such as module completion or time spent in specific components, would be needed to allow a detailed dose-response analysis. This was not feasible with available data. In particular, for unguided digital interventions such as the present one, adherence and usage behavior warrant a critical view 36 , as patients may discontinue use or engage with the app in ways that deviate from the intended protocol. A blended care environment may enhance adherence and thus the efficacy of digital interventions by incorporating brief feedback loops or minimal guidance. It should therefore be considered in future studies and routine implementation.

### Clinical Implications and Conclusion

The findings of this study highlight the potential of comprehensive, app-based interventions like the Mika-App to complement standard psychosocial care for patients with cancer by providing accessible, scalable and low-threshold support. In our sample, we did not find a moderation by age and DHL , thus questioning common assumptions about digital health use among older adults. Clinicians might consider recommending digital support tools not only to younger, digitally literate patients. However, to ensure equitable access, further research is needed to identify and address barriers related to DHL and socioeconomic factors in a real-world clinical sample derived from routine hospital care settings. Incorporating digital support tools into clinical workflows may empower patients to actively manage their psychological well-being throughout the cancer trajectory.

## Fundref Information

Fosanis GmbH, Berlin

## Ethics

The study protocol was approved by the ethics committee of the Medical Faculty of Leipzig University (404/21-ek), Leipzig, Germany. All participants gave informed consent prior to study enrollment.

## Data availability statement

The data sets generated and analyzed during this study are available from AMT after 3 months and up to 5 years following the publication of the article. Access to the data will be granted to investigators whose proposed use of the data has been approved by an independent review committee identified for this purpose, for individual patient data meta-analysis. Proposals for accessing the data may be submitted up to 36 months following article publication.

## Author contribution

Conception and design: AMT, FS, JSR Financial support: JSR, GF Administrative support: FS, HB Collection and assembly of data: FS Data analysis and interpretation: DK, FS, HB Supervision: AMT Writing original draft: FS Review, editing and final approval of manuscript: All authors

## Declaration of generative AI and AI-assisted technologies in the writing process

During the preparation of this work the authors used DeepL in order to improve language and readability. After using this tool, the authors reviewed and edited the content as needed and take full responsibility for the content of the publication.
